# Differentiation of retropharyngeal calcific tendinitis and retropharyngeal abscess: a case series and review of the literature

**DOI:** 10.1007/s00405-020-06057-w

**Published:** 2020-05-24

**Authors:** Soenke Langner, Christian Ginzkey, Robert Mlynski, Nora M. Weiss

**Affiliations:** 1 Department of Radiology, Rostock University Medical Center, Schillingallee 35, 18057 Rostock, Germany; 2 Department of Otorhinolaryngology, Head and Neck Surgery “Otto Körner”, Rostock University Medical Center, Doberaner Strasse 137-139, 18057 Rostock, Germany

**Keywords:** Prevertebral tendinitis, Pain, Neck, Neck stiffness

## Abstract

**Introduction:**

Retropharyngeal calcific tendinitis (RCT) is a self-limiting aseptic inflammation of the tendon of the longus colli muscle, which can be clinically and radiologically misdiagnosed as abscess formation. This is a particular challenge for ENT specialists. However, articles about RCT are highly underrepresented in ENT journals and existing articles in ENT journals almost exclusively report overtreatment.

**Methods:**

This study presents five patients, in which the diagnosis of RCT was delayed and of which one patient underwent incision and draining of a suspected retropharyngeal abscess under general anesthesia. In addition, the literature on the reported cases of RCT, between 1990 and 2020 was reviewed. For each case, epidemiological characteristics, complaints on presentation, symptoms, imaging and laboratory finding and treatment were summarized and compared to our own findings.

**Results:**

In all the five patients, the correct diagnosis was delayed. One patient underwent incision and draining of a suspected RA under general anesthesia. All patients received antibiotic treatment. The literature review revealed a total of 116 reported cases of RCT. A total of 99 CT scans and 72 MRI showed soft tissue swelling in 89.6% and calcifications in 91.4% of the cases, 6.9% received invasive treatment.

**Conclusion:**

This article emphasizes the importance of knowledge about RCT and its management to avoid invasive and potentially harmful treatment. The focus in establishing the correct diagnosis of RCT is the identification and correct interpretation of clinical symptoms together with the specific radiological findings.

**Electronic supplementary material:**

The online version of this article (10.1007/s00405-020-06057-w) contains supplementary material, which is available to authorized users.

## Introduction

Retropharyngeal calcific tendinitis (RCT) is an aseptic inflammation of the tendon of the longus colli muscle (LCM). The superior oblique portion is considered to be the origin of the inflammation [1, 2]. Hartley described the first case of RCT in 1964 [3]. The cause of the disease is not completely understood. A possible pathological correlate is the deposit of calcium hydroxyapatite crystals in the fascia of the LCM [2]. It has been suggested that these deposits result from macrophage activation due to chronic tendinitis following microtraumata [4]. The calcification can lead to an antibody reaction and to edema around the vertebral bodies C1–4 [5]. Common symptoms are odynophagia, neck stiffness and limited movement of the neck without severely increased white blood-cell-count (WBC) or fever [2]. RCT typically affects adults aged between 30 and 60 years [3]. The constellation of symptoms usually leads to a consultation of either an emergency unit or an ear, nose and throat (ENT) specialist. Clinical examination frequently does not show severe findings, such as trismus, edema of the soft palate, pharyngeal swelling or fever. Radiologic imaging is crucial in establishing the diagnosis and reveals soft tissue swelling in the retropharyngeal space and calcifications of the LCM [6]. The course of RCT is self-limiting and invasive treatment should be avoided. Therapy of RCT consists of analgesia with non-steroid antiphlogistic drugs (NSAID) and can be combined with corticosteroids [2, 5]. Due to the rare incidence of RCT, the literature almost exclusively consists of case reports. Awareness of RCT is important since severe or life-threatening differential diagnoses such as retropharyngeal abscess (RA), meningitis or spondylodiscitis are much more common and need to be treated differently, including antibiotic treatment and/or surgery. Since the symptoms of RCT are rather unspecific and due to the self-limiting course, the frequency of RCT might be underestimated and the incidence may be higher than initially estimated [7].

This article presents a case series of five patients with RCT, illustrates the risk of misdiagnosis and adds a detailed review of the literature analyzing the treatment and symptoms of a large number of RCT.

## Methods

### Patient selection

We report a case series with a delayed diagnosis of RCT who were treated at our department of otolaryngology, head and neck surgery at a tertiary hospital between 2015 and 2020.

Written informed consent was obtained from all patients.

### Literature review

Literature search was performed on PubMed on search terms “Prevertebral tendinitis”, “Retropharyngeal calcific tendinitis” and “Acute retropharyngeal calcific tendinitis” (see supplementary document “RCT_Review” in Supporting Information for detailed search strategy).

### Inclusion/exclusion criteria

Case series and reports reporting radiographic (CT or MRI) and clinical examination findings were included. Publications with isolated radiological information lacking details on clinical findings, publications reporting X-ray diagnostics only, and publications in languages other than English and German were excluded.

## Results

### Own cases

**Fig. 1:**
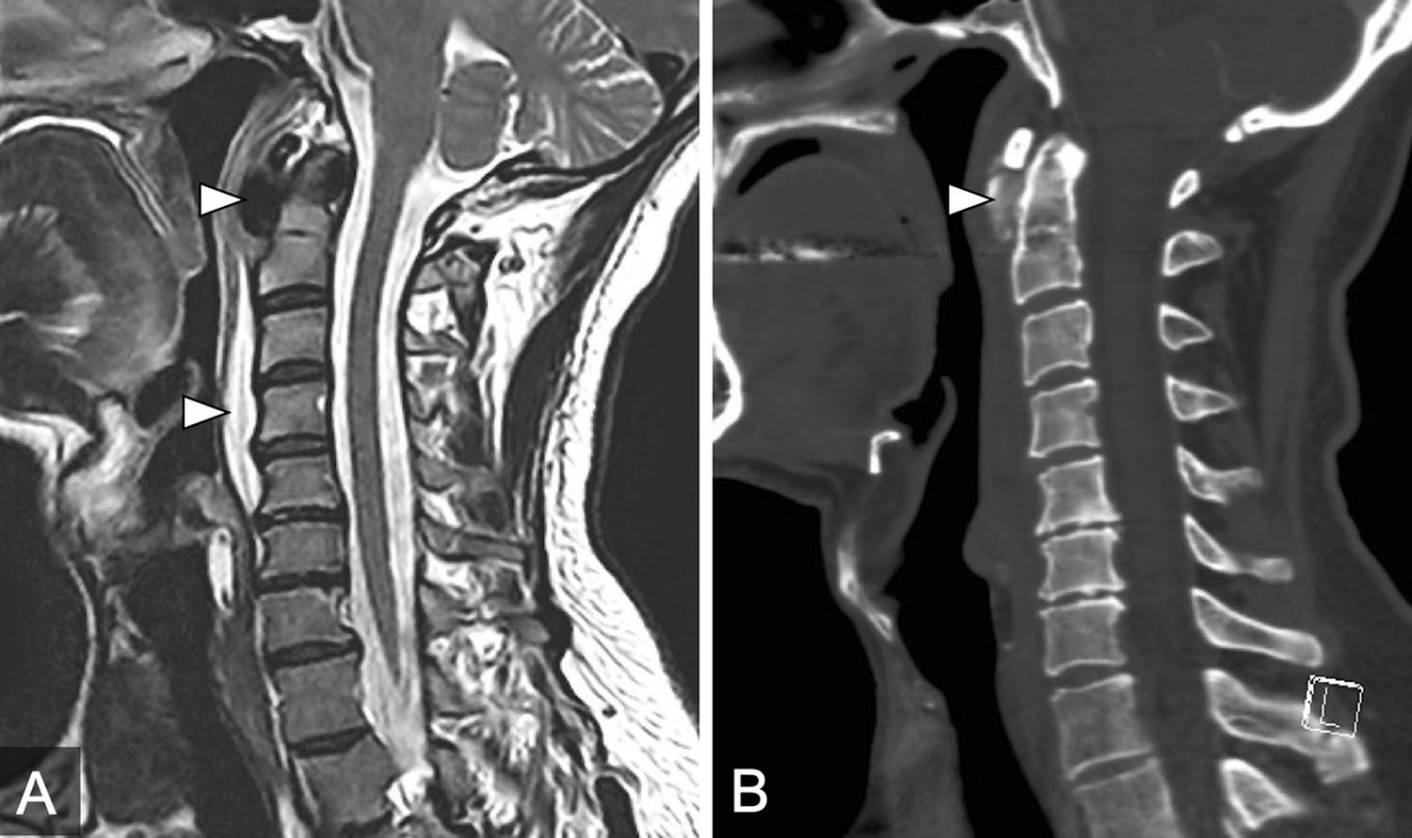
MRI and CT of a 56-year-old male patient with acute neck pain and impairment of neck movement (Patient 1). **a** Sagittal T2-weigthed image demonstrating prevertebral calcification at the level of C1/C2 (upper white arrowhead) with adjacent long sectional edematous prevertebral infiltration from C2–C5 (lower white arrowhead). Due to the diagnosis of a RA on the initial MRI, which was performed on an outpatient basis, he received intravenous antibiotic and analgetic therapy (Table 1). **b** Sagittal reconstruction of a CT scan in bone window setting demonstrating coarse prevertebral calcification at the level C1/C2 (white arrowhead). This follow-up CT was performed one day after treatment initiation. After the initiation of antibiotic and analgetic treatment, symptoms resolved within one week

**Fig. 2:**
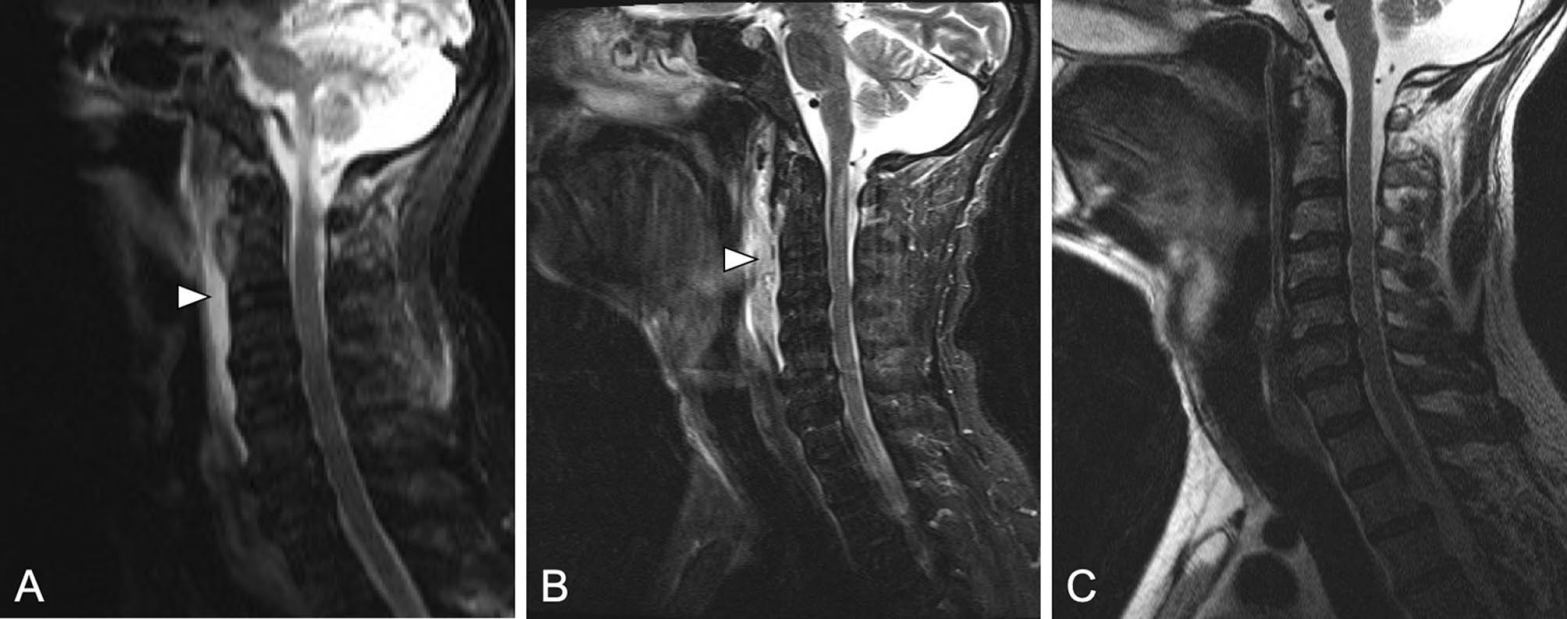
MRI of a 55-year-old male patient presenting with a 2-week history of pain when moving his neck with acute limitation of neck mobility (Patient 2). Upon clinical examination including transnasal fiberoptic endoscopy of the pharynx and larynx, no visible swelling was noted. **a** Sagittal T2-weighted MR image with fat saturation of the cervical spine showing extensive prevertebral edema at the level of C1–C6 (white arrowhead) and discrete joint effusion of the atlanto-axial joint leading to the diagnosis of RA/septic arthritis. Subsequently, the patient underwent a transcervical exploration of the retropharyngeal space. However, intraoperatively, no abscess formation was found. Six hours after surgery, the patient suffered from a postoperative bleeding from the surgical field that made the revision surgery necessary. As a complication, the patient suffered from a postoperative hypoglossal nerve paresis. **b** Sagittal T2-weightend MR image with fat saturation 5 days postoperatively showing increased effusion with postoperative edema (white arrowhead). At this time, neck pain had decreased under antibiotic and analgetic treatment. **c** Sagittal T2-weightend MR image with fat saturation 6 weeks postoperatively showing complete resolution of MRI findings

**Fig. 3:**
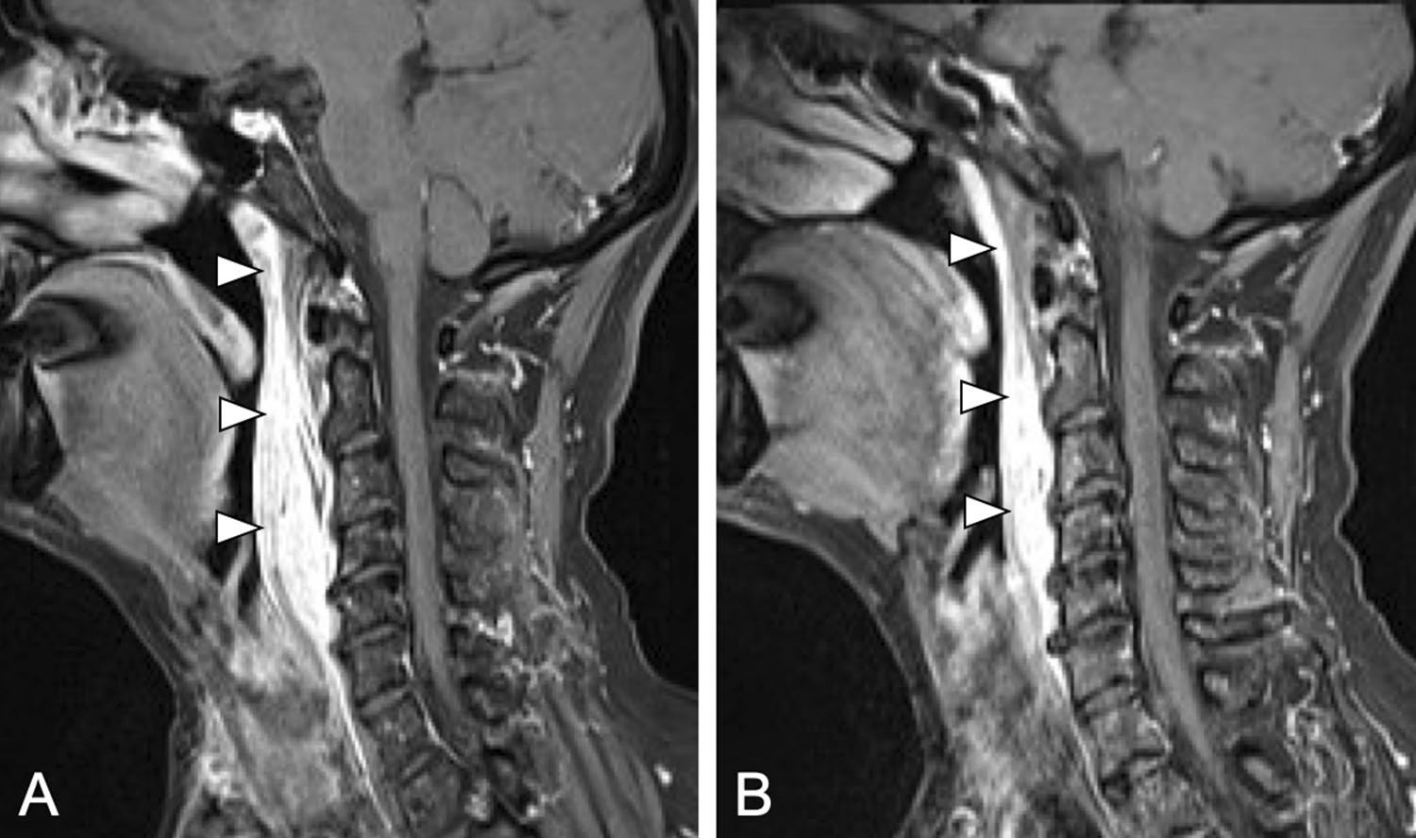
Short-term remission of MRI findings in a 55-year-old female patient (Patient 3) initially presenting at another hospital with fever and tachycardia, who was treated with oral antibiotics. During the course of treatment, she developed progressive odynophagia. On clinical presentation at our institution, symptoms had already decreased. **a** Sagittal T1-weighted image with fat saturation after contrast agent administration of the initial MRI performed on the day of admission with large edema in the retropharyngeal space at level C1–C5/6 (white arrowheads). RCT was diagnosed and a conservative treatment was initiated. **b** Sagittal T1-weighted follow-up MRI with fat saturation 8 days after initiation of the treatment showing decreased inflammatory infiltrations (white arrowheads). By that time, the patient was asymptomatic

Five patients with RCT admitted to our department were identified. Details on demographics, laboratory findings and treatment of each individual patient are summarized in Table 1. Selected patients are presented in Figs. 1, 2, 3.

**Table 1:** Patient demographics and treatment including the literature review

	Reviewed data (*n* = 116)	Own data (*n* = 5)
Mean age—years	46.0 (SD 11.0)	55.4 (SD 1.8)
Sex (m:f)—*n* (%)	52:64 (45:55)	3:2 (60:40)
Symptoms—*n* (%)		
Neck pain	108 (93.1)	5 (100)
Neck stiffness	94 (81.0)	5 (100)
Dysphagia/odynophagia	92 (79.3)	2 (40)
Limitation of neck movement	59 (50.8)	4 (80)
Laboratory results		
Mean WBC–10^9^/l	10.6 (SD 2.3)	10.3 (SD 2.4)
Mean CRP–mg/l	7.7 (SD 13.9)	36.9 (SD 50.4)
Temperature >38°C/100.4°F–*n* (%)	4 (3.4)	0 (0)
Imaging modality—*n* (%)		
MRI	72 (62.0)	5 (100)
CT	99 (85.3)	3 (60)
Imaging findings—*n* (%)		
Soft tissue swelling	104 (89.6)	5 (100)
Prevertebral calcification	106 (91.4)	4 (80)
Prevertebral effusion	91 (78.4)	5 (100)
Treatment—*n* (%)		
NSAID	101 (87.1)	2 (40)
Antibiotics	36 (31.0)	4 (80)
Corticosteroids	33 (28.4)	2 (40)
Soft collar	11 (9.5)	0 (0)
Needle aspiration/surgical treatment	8 (6.9)	1 (20)

### Literature review

Literature review revealed 58 articles, of which 42 were single case reports and 16 were case series with two to ten patients. In all cases, RCT was diagnosed by clinical examination and additional tomography. Of these articles, 14 were published in radiology journals, 13 in orthopedic or rheumatologic journals, 5 in emergency medicine journals, 4 in neurology and pain journals, 7 in internal medicine journals and 6 in ENT journals. Nine articles were published in other journal types. In total, 116 cases were reviewed. A summary of demographics, complaints on presentation, symptoms, imaging and laboratory findings and treatment of these patients is given in Table 1.

## Discussion

Retropharyngeal calcific tendinitis (RCT) is a rare disease and may be difficult to diagnose. Besides clinical and laboratory diagnostic results, tomography is needed to confirm RCT and exclude potentially harmful differential diagnoses. Due to its clinical appearance with neck stiffness, painful retroflexion of the neck, subfebrile body temperature, odynophagia and occurrence of pharyngeal edema, RCT can mimic RA and may easily be misdiagnosed with the consequence of overtreatment [8]. In addition, a thickening of the retropharyngeal space with raised fluid content may be radiologically misdiagnosed as abscess formation. From our patient cohort, four patients (80%) received intravenous antibiotics, two patients (40%) were treated with corticosteroids and one patient (20%) had invasive surgical treatment. Compared to the literature review in which only 31% of the patients received antibiotics, this rate is exceptionally high. Reasons for this discrepancy in treatment regimens may be the different clinical specialties treating these patients. While the majority of papers on RCT were published in journals for emergency medicine, radiology or orthopedics, such reports were rarely published in ENT journals. Whereas radiologists can strictly focus on typical imaging findings of the disease, we assume that for ENT specialists, it is hard to decide against antibiotic treatment, when a patient presents with the typical symptoms of the well-known diagnosis of RA, despite laboratory findings being not indicative for infectious disease. Hammer et al. published data from ten patients in an ENT journal, where 50% of the patients were treated with antibiotics under the initial diagnosis of RA [8]. Another publication from an ENT journal presents a comparatively high incidence of antibiotic treatment in 6/8 (75%) patients [7] confirming the assumption that antibiotic treatment is rather overrepresented when the patient first presents to an ENT specialist. A patients similar to our patient 2, in which surgical intervention without intraoperative findings confirming RA was performed, was also reported by a group of ENT specialists [9]. ENT specialists presumably tend to choose a treatment option giving the safety of antibacterial treatment comparing the low risk of side effects to the life-threatening consequences of insufficient treatment of common bacterial infections of the head and neck. Considering complications due to overtreatment as exemplified in patient 2 in our series suffering from postoperative hypoglossal nerve paresis, we emphasize the importance of correct radiologic diagnosis to avoid unnecessary invasive treatment. In contrast to the therapy of RCT [2], RA has to be immediately treated with a combination of intravenous antibiotics, sufficient analgesia and surgery. Nevertheless, considering the life-threatening complications of differential diagnoses, such as an abscess, is mandatory. Any fluid formation in the cervical soft tissues is a potential substrate for secondary bacterial infection. Additionally, co-morbidities associated with immunodeficiency justify the individual treatment with antibiotics.

Approaches to distinguish between RCT and RA or other differential diagnoses, such as spondylodiscitis, based on clinical presentation and laboratory results seem to be successful [8]. While only mild elevation of WBC, limitation of neck movement and pain are unspecific and cannot differentiate between RCT and RA, endoscopic findings visualizing potential edema of the mucosa are crucial in the clinical differentiation between RCT and RA. Trismus, mucosal edema and asymmetry of the pharyngeal space are clinical signs for an abscess and can only be seen during clinical and endoscopic examination.

Because of the equivocal clinical findings, RCT often is considered an imaging diagnosis [1, 6]. On plain radiographs, the typical imaging finding for both RA and RCT is swelling of the prevertebral space. On CT, prevertebral fluid collections and swelling of the LCM can be identified and calcifications in the superior part of the muscle are considered to be pathognomonic. Therefore, CT is considered to be the imaging modality of choice in cases of suspected RCT [1, 2, 6]. On T2-weighted MR images, edematous changes of the prevertebral muscles present as signal increase and after contrast administration, the affected muscle and the surrounding tissue demonstrate avid enhancement. Diffusion-weighted imaging may facilitate the differential diagnosis to RA formation [8, 10]. While coarse calcification can be seen on MRI, fine-granulated calcifications are more easily detected on CT [10]. Therefore, CT and MRI should be considered complementary in the diagnostic work-up of these patients. A correct interpretation of the radiologic changes by an experienced radiologist is essential, since tomography imaging is mandatory to confirm the diagnosis and to determine further therapy [1, 2, 6, 10]. Yet, only the combination of clinical, endoscopic and laboratory findings with those made in MRI or CT can lead to the correct diagnosis and to a safe treatment for the patient.

Although this study is limited by a small number of cases with inconsistent therapeutic assessment, we believe that this constellation realistically depicts the obstacles arising through the rare diagnosis of RCT. This article aims to point out pitfalls in clinical routine and strengthen the awareness of RCT.

## Conclusion

RCT is a rare disease that can be misdiagnosed as a severe inflammation and therefore be overtreated with invasive procedures. It is particularly demanding for ENT specialists not to overestimate the clinical findings and misdiagnose severe infections, which need to be treated with antibiotics and surgical intervention. An interdisciplinary approach is important for ENT specialists and radiologists to be aware of RCT with its typical clinical symptoms, such as sore throat and neck stiffness, and key radiological finding, i.e. prevertebral calcifications.

## Electronic supplementary material

Below is the link to the electronic supplementary material.

Details on performed RCT literature review, including single references and search strategy (DOCX 36 kb)
