# Supplementary material for: Differentiation of retropharyngeal calcific tendinitis and retropharyngeal abscess: a case series and review of the literature
Source: Eur Arch Otorhinolaryngol. 2020 May 24;277(9):2631–6. doi: 10.1007/s00405-020-06057-w (PMC7410824; doi:10.1007/s00405-020-06057-w)
Supplement: Supplementary file 1 — Details on performed RCT literature review, including single references and search strategy (DOCX 36 kb) [file 405_2020_6057_MOESM1_ESM.docx]

**1: Naik PP, Savery N, Kuruvilla L, Nayakar G, Raghul T. Longus Colli Tendinitis:**

**The Lost Twin of Retropharyngeal Abscess. Indian J Otolaryngol Head Neck Surg.**

**2019 Oct;71(Suppl 1):771-775. doi: 10.1007/s12070-018-1541-y. Epub 2018 Nov 30.**

**PubMed PMID: 31742062; PubMed Central PMCID: PMC6848692.**

**2: Frei N, Bless N. Acute Longus Colli Calcific Tendinitis: A Rare Cause of Neck**

**Pain. Eur J Case Rep Intern Med. 2019 Aug 2;6(8):001126. doi:**

**10.12890/2019_001126. eCollection 2019. PubMed PMID: 31508382; PubMed Central**

**PMCID: PMC6726343.**

**3: Matsuura H, Sugimoto Y, Sasaki E, Kiura Y, Kishida M. Acute calcific**

**retropharyngeal tendinitis. Postgrad Med J. 2019 Dec;95(1130):678. doi:**

**10.1136/postgradmedj-2019-136750. Epub 2019 Jul 30. PubMed PMID: 31363017.**

**4: Munson L, Funk MF, Perrault TA. Retropharyngeal Calcific Tendonitis in a**

**Patient Seeking Chiropractic Care for Neck Pain: A Case Report. J Chiropr Med.**

**2018 Dec;17(4):275-282. doi: 10.1016/j.jcm.2018.04.006. Epub 2019 Feb 25. PubMed**

**PMID: 30846921; PubMed Central PMCID: PMC6391230.**

**5: Zipfel N, Schlüter A, Keyßer G, Schäfer C. [A 34-year-old patient with**

**retropharyngeal tendinitis]. Z Rheumatol. 2019 Jun;78(5):458-460. doi:**

**10.1007/s00393-019-0620-0. German. PubMed PMID: 30838437.**

**6: Nakagami F, Hagiya H, Rakugi H. Acute Non-calcific Retropharyngeal Tendinitis.**

**Intern Med. 2018 Dec 1;57(23):3499-3500. doi: 10.2169/internalmedicine.1127-18.**

**Epub 2018 Aug 10. PubMed PMID: 30101923; PubMed Central PMCID: PMC6306551.**

**7: Bhatt AA. Non-traumatic causes of fluid in the retropharyngeal space. Emerg**

**Radiol. 2018 Oct;25(5):547-551. doi: 10.1007/s10140-018-1619-6. Epub 2018 Jun 23.**

**Review. PubMed PMID: 29934925.**

**8: Suh B, Eoh J, Shin J. Clinical and Imaging Features of Longus Colli Calcific**

**Tendinitis: An Analysis of Ten Cases. Clin Orthop Surg. 2018 Jun;10(2):204-209.**

**doi: 10.4055/cios.2018.10.2.204. Epub 2018 May 18. PubMed PMID: 29854344; PubMed**

**Central PMCID: PMC5964269.**

82: Shen Y, Zhou Q, Zhu X, Qiu Z, Jia Y, Liu Z, Li S. Vertigo caused by longus

colli tendonitis: A case report and literature review. Medicine (Baltimore). 2018

Nov;97(45):e13130. doi: 10.1097/MD.0000000000013130. Review. PubMed PMID:

30407336; PubMed Central PMCID: PMC6250553.

4: Uchida T, Kanzaki M, Kakumoto T, Uesaka Y. Longus Colli Tendinitis in a

Patient Presenting with Neck Pain and Acute Systemic Inflammation. Intern Med.

2018 Sep 15;57(18):2759-2761. doi: 10.2169/internalmedicine.0160-17. Epub 2018

Apr 27. PubMed PMID: 29709928; PubMed Central PMCID: PMC6191587.

**10: Raggio BS, Ficenec SC, Pou J, Moore B. Acute Calcific Tendonitis of the**

**Longus Colli. Ochsner J. 2018 Spring;18(1):98-100. PubMed PMID: 29559880; PubMed**

**Central PMCID: PMC5855435.**

**11: Kim YJ, Park JY, Choi KY, Moon BJ, Lee JK. Case reports about an overlooked**

**cause of neck pain: calcific tendinitis of the longus colli: Case reports.**

**Medicine (Baltimore). 2017 Nov;96(46):e8343. doi: 10.1097/MD.0000000000008343.**

**PubMed PMID: 29145245; PubMed Central PMCID: PMC5704790.**

**12: Alamoudi U, Al-Sayed AA, AlSallumi Y, Rigby MH, Taylor SM, Hart RD, Trites**

**JRB. Acute calcific tendinitis of the longus colli muscle masquerading as a**

**retropharyngeal abscess: A case report and review of the literature. Int J Surg**

**Case Rep. 2017;41:343-346. doi: 10.1016/j.ijscr.2017.10.063. Epub 2017 Nov 11.**

**PubMed PMID: 29145108; PubMed Central PMCID: PMC5686463.**

**13: Abdelbaki A, Abdelbaki S, Bhatt N, Gupta N, Li S, Ghasemiesfe A, Kumar Y.**

**Acute Calcific Tendinitis of the Longus Colli Muscle: Report of Two Cases and**

**Review of the Literature. Cureus. 2017 Aug 23;9(8):e1597. doi:**

**10.7759/cureus.1597. PubMed PMID: 29067221; PubMed Central PMCID: PMC5652888.**

**14: Naik PP, Poduval J, Divakaran S. Review Article: Retropharyngeal**

**Abscess-Mimickers and Masqueraders. Indian J Otolaryngol Head Neck Surg. 2017**

**Jun;69(2):269-273. doi: 10.1007/s12070-017-1105-6. Epub 2017 Feb 28. Erratum in:**

**Indian J Otolaryngol Head Neck Surg. 2017 Jun;69(2):274-275. PubMed PMID:**

**28607904; PubMed Central PMCID: PMC5446347.**

**15: Abou Dargham H, Bytyci F, Shuman C, Stolear A. A rare cause of acute**

**dysphagia: acute calcific tendonitis of the longus colli muscle. BMJ Case Rep.**

**2017 May 27;2017. pii: bcr-2017-219684. doi: 10.1136/bcr-2017-219684. PubMed**

**PMID: 28551599; PubMed Central PMCID: PMC5612563.**

8: Boardman J, Kanal E, Aldred P, Boonsiri J, Nworgu C, Zhang F. Frequency of

acute longus colli tendinitis on CT examinations. Emerg Radiol. 2017

Dec;24(6):645-651. doi: 10.1007/s10140-017-1537-z. Epub 2017 Jul 25. PubMed PMID:

28744692; PubMed Central PMCID: PMC5681978.

9: Kenzaka T, Kumabe A. Acute Calcific Prevertebral Tendinitis. Intern Med.

2017;56(12):1611. doi: 10.2169/internalmedicine.56.8266. Epub 2017 Jun 15. PubMed

PMID: 28626196; PubMed Central PMCID: PMC5505926.

11: Shibuki T, Goto H, Fukushima K, Mizuta T. Acute Prevertebral Calcific

Tendinitis. Intern Med. 2017;56(10):1275. doi: 10.2169/internalmedicine.56.8197.

Epub 2017 May 15. PubMed PMID: 28502954; PubMed Central PMCID: PMC5491834.

**16: Patel TK, Weis JC. Acute neck pain in the ED: Consider longus colli calcific**

**tendinitis vs meningitis. Am J Emerg Med. 2017 Jun;35(6):943.e3-943.e4. doi:**

**10.1016/j.ajem.2017.01.055. Epub 2017 Jan 25. PubMed PMID: 28139306.**

12: Simões J, Romão J, Cunha A, Paiva S, Miguéis A. Neck Pain and Acute

Dysphagia. Dysphagia. 2017 Feb;32(1):123-125. doi: 10.1007/s00455-016-9764-0.

Epub 2016 Nov 30. PubMed PMID: 27904957.

**17: Zapolsky N, Heller M, Felberbaum M, Rose J, Steinberg E. Calcific Tendonitis**

**of the Longus Colli: An Uncommon but Benign Cause of Throat Pain that Closely**

**Mimics Retropharyngeal Abscess. J Emerg Med. 2017 Mar;52(3):358-360. doi:**

**10.1016/j.jemermed.2016.08.016. Epub 2016 Oct 17. PubMed PMID: 27765436.**

**18: Yamashita Y, Sakuma Y, Sibata K, Komatsu M, Niwa K, Takada K, Kuwahara T,**

**Kasai N, Sagou T, Takayanagi H, Oridate N. Three Cases of Acute Calcific**

**Retropharyngeal Tendinitis. Nihon Jibiinkoka Gakkai Kaiho. 2016**

**Jul;119(7):955-61. Japanese. PubMed PMID: 30051977.**

**19: Tamm A, Jeffery CC, Ansari K, Naik S. Acute Prevertebral Calcific Tendinitis.**

**J Radiol Case Rep. 2015 Nov 30;9(11):1-5. doi: 10.3941/jrcr.v9i11.2494.**

**eCollection 2015 Nov. PubMed PMID: 27252789; PubMed Central PMCID: PMC4871136.**

**20: Tomita H, Yamashiro T, Ikeda H, Fujikawa A, Kurihara Y, Nakajima Y. Fluid**

**collection in the retropharyngeal space: A wide spectrum of various emergency**

**diseases. Eur J Radiol. 2016 Jul;85(7):1247-56. doi: 10.1016/j.ejrad.2016.04.001.**

**Epub 2016 Apr 14. Review. PubMed PMID: 27235871.**

**21: Oh JY, Lim JH, Kim YS, Kwon YE, Yu JY, Lee JH. Misconceived Retropharyngeal**

**Calcific Tendinitis during Management of Myofascial Neck Pain Syndrome. Korean J**

**Pain. 2016 Jan;29(1):48-52. doi: 10.3344/kjp.2016.29.1.48. Epub 2016 Jan 4.**

**PubMed PMID: 26839671; PubMed Central PMCID: PMC4731552.**

**22: Nozu T, Kumei S, Ohhira M, Okumura T. Retropharyngeal Calcific Tendinitis.**

**Intern Med. 2015;54(17):2277. doi: 10.2169/internalmedicine.54.4682. Epub 2015**

**Sep 1. PubMed PMID: 26328662.**

**23: Yaylacı S, Öztürk TC, Aksoy E, Koçyigit A, Yılmaz A, Karaarslan E.**

**Retropharyngeal calcific tendinitis: Report of two cases. J Emerg Trauma Shock.**

**2015 Apr-Jun;8(2):119-20. doi: 10.4103/0974-2700.145408. PubMed PMID: 25949045;**

**PubMed Central PMCID: PMC4411574.**

**24: Tagashira Y, Watanuki S. Acute calcific retropharyngeal tendonitis. CMAJ.**

**2015 Sep 22;187(13):995. doi: 10.1503/cmaj.140214. Epub 2015 Apr 27. PubMed PMID:**

**25918175; PubMed Central PMCID: PMC4577348.**

**25: Bailey CW, Connolly BD, Sterner K, Roberts TD. Acute calcific longus colli**

**tendonitis. W V Med J. 2015 Mar-Apr;111(2):10-2. PubMed PMID: 25842699.**

**26: Estimable K, Rizk C, Pujalte GG. A rare case of neck pain: acute longus colli**

**calcific tendinitis in a possibly immunocompromised individual. J Am Board Fam**

**Med. 2015 Jan-Feb;28(1):146-50. doi: 10.3122/jabfm.2015.01.140124. PubMed PMID:**

**25567835.**

**27: Chen CH, Lu YC, Wong TY. Acute calcific prevertebral tendinitis: rare cause**

**of neck pain. Acute Med Surg. 2014 Nov 21;2(3):199-201. doi: 10.1002/ams2.92.**

**eCollection 2015 Jul. PubMed PMID: 29123721; PubMed Central PMCID: PMC5667259.**

**28: Tezuka F, Sakai T, Miyagi R, Takata Y, Higashino K, Katoh S, Sairyo K, Yasui**

**N. Complete resolution of a case of calcific tendinitis of the longus colli with**

**conservative treatment. Asian Spine J. 2014 Oct;8(5):675-9. doi:**

**10.4184/asj.2014.8.5.675. Epub 2014 Oct 18. PubMed PMID: 25346822; PubMed Central**

**PMCID: PMC4206819.**

**29: Johari S, Handa P, Siow JK. Retropharyngeal pseudoabscess manifesting in**

**nephrotic syndrome. Ear Nose Throat J. 2014 Apr-May;93(4-5):E27-9. PubMed PMID:**

**24817238.**

**30: Ohtsuka Y, Chazono H, Suzuki H, Ohkuma Y, Sakurai T, Hanazawa T, Okamoto Y.**

**[Eight cases of calcific retropharyngeal tendinitis/retropharyngeal calcific**

**tendinitis]. Nihon Jibiinkoka Gakkai Kaiho. 2013 Nov;116(11):1200-7. Japanese.**

**PubMed PMID: 24397117.**

**31: Silva CF, Soffia PS, Pruzzo E. Acute prevertebral calcific tendinitis: a**

**source of non-surgical acute cervical pain. Acta Radiol. 2014 Feb;55(1):91-4.**

**doi: 10.1177/0284185113492151. Epub 2013 Jul 17. PubMed PMID: 23864065.**

**32: Gabra N, Belair M, Ayad T. Retropharyngeal calcific tendinitis mimicking a**

**retropharyngeal phlegmon. Case Rep Otolaryngol. 2013;2013:912628. doi:**

**10.1155/2013/912628. Epub 2013 Jun 3. PubMed PMID: 23862089; PubMed Central**

**PMCID: PMC3686063.**

**33: Trendel D, Bonfort G, Lapierre-Combes M, Salf E, Barberot JP. Acute severe**

**neck pain and dysphagia following cervical maneuver: diagnostic approach. Eur Ann**

**Otorhinolaryngol Head Neck Dis. 2014 Apr;131(2):135-8. doi:**

**10.1016/j.anorl.2013.03.002. Epub 2013 Jul 9. PubMed PMID: 23845292.**

**34: Horowitz G, Ben-Ari O, Brenner A, Fliss DM, Wasserzug O. Incidence of**

**retropharyngeal calcific tendinitis (longus colli tendinitis) in the general**

**population. Otolaryngol Head Neck Surg. 2013 Jun;148(6):955-8. doi:**

**10.1177/0194599813482289. Epub 2013 Mar 22. PubMed PMID: 23525848.**

**35: Terao K, Kusunoki T, Mori K, Murata K, Doi K. A case of calcific**

**retropharyngeal tendinitis: the significance of an early diagnosis. Ear Nose**

**Throat J. 2013 Feb;92(2):74-83. PubMed PMID: 23460215.**

**36: Carcia CR, Scibek JS. Causation and management of calcific tendonitis and**

**periarthritis. Curr Opin Rheumatol. 2013 Mar;25(2):204-9. doi:**

**10.1097/BOR.0b013e32835d4e85. Review. PubMed PMID: 23370373.**

**37: Desmots F, Derkenne R, Couppey N, Martin B, Gabaudan C, Geffroy Y. Calcific**

**retropharyngeal tendinitis of the longus colli muscle. Answer to the e-quid**

**"Acute cervical pain and dysphagia in a 43 year-old man". Diagn Interv Imaging.**

**2013 Apr;94(4):470-3. doi: 10.1016/j.diii.2012.09.006. Epub 2013 Jan 26. PubMed**

**PMID: 23357277.**

**38: Zibis AH, Giannis D, Malizos KN, Kitsioulis P, Arvanitis DL. Acute calcific**

**tendinitis of the longus colli muscle: case report and review of the literature.**

**Eur Spine J. 2013 May;22 Suppl 3:S434-8. doi: 10.1007/s00586-012-2584-5. Epub**

**2012 Nov 21. Review. PubMed PMID: 23179983; PubMed Central PMCID: PMC3641270.**

**39: Boikov AS, Griffith B, Stemer M, Jain R. Acute calcific longus colli**

**tendinitis: an unusual location and presentation. Arch Otolaryngol Head Neck**

**Surg. 2012 Jul;138(7):676-9. doi: 10.1001/archoto.2012.910. PubMed PMID:**

**22801892.**

**40: Ahmed OH, German MA, Handwerker J, Bhandarkar ND. Radiology quiz case 2.**

**Acute calcific tendinitis of the longus colli (also known as calcific**

**retropharyngeal/prevertebral tendinitis). Arch Otolaryngol Head Neck Surg. 2012**

**Jun;138(6):599-600. doi: 10.1001/archoto.2012.519a 10.1001/archoto.2012.519b.**

**PubMed PMID: 22710516.**

**41: Pope LE, McHale E, Pretorius PM, Bottrill I. Radiology quiz case 1. Calcific**

**prevertebral tendinitis (also known as calcific retropharyngeal tendinitis and**

**calcific tendinitis of the longus colli). Arch Otolaryngol Head Neck Surg. 2011**

**Sep;137(9):953, 954. doi: 10.1001/archoto.2011.148-a. PubMed PMID: 21930989.**

**42: Lee S, Joo KB, Lee KH, Uhm WS. Acute retropharyngeal calcific tendinitis in**

**an unusual location: a case report in a patient with rheumatoid arthritis and**

**atlantoaxial subluxation. Korean J Radiol. 2011 Jul-Aug;12(4):504-9. doi:**

**10.3348/kjr.2011.12.4.504. Epub 2011 Jul 22. PubMed PMID: 21852912; PubMed**

**Central PMCID: PMC3150679.**

**43: Aydil U, Kizil Y, Köybaşioğlu A. Less known non-infectious and**

**neuromusculoskeletal system-originated anterolateral neck and craniofacial pain**

**disorders. Eur Arch Otorhinolaryngol. 2012 Jan;269(1):9-16. doi:**

**10.1007/s00405-011-1746-0. Epub 2011 Aug 13. Review. PubMed PMID: 21842201.**

**44: Coulier B, Macsim M, Desgain O. Retropharyngeal calcific tendinitis--longus**

**colli tendinitis--an unusual cause of acute dysphagia. Emerg Radiol. 2011**

**Oct;18(5):449-51. doi: 10.1007/s10140-011-0959-2. Epub 2011 May 27. PubMed PMID:**

**21617936.**

**45: Martindale JL, Senecal EL. Atraumatic neck pain and rigidity: a case of**

**calcific retropharyngeal tendonitis. Am J Emerg Med. 2012 May;30(4):636.e1-2.**

**doi: 10.1016/j.ajem.2011.02.004. Epub 2011 Apr 22. PubMed PMID: 21514765.**

**46: Queinnec S, Petrover D, Guigui P, Ilharreborde B. Benign febrile cervicalgia**

**due to calcific retropharyngeal tendinitis: case study. Orthop Traumatol Surg**

**Res. 2011 May;97(3):341-4. doi: 10.1016/j.otsr.2010.09.021. Epub 2011 Mar 31.**

**PubMed PMID: 21458398.**

**47: Shin DE, Ahn CS, Choi JP. The acute calcific prevertebral tendinitis: report**

**of two cases. Asian Spine J. 2010 Dec;4(2):123-7. doi: 10.4184/asj.2010.4.2.123.**

**Epub 2010 Nov 24. PubMed PMID: 21165316; PubMed Central PMCID: PMC2996624.**

**48: Park SY, Jin W, Lee SH, Park JS, Yang DM, Ryu KN. Acute retropharyngeal**

**calcific tendinitis: a case report with unusual location of calcification.**

**Skeletal Radiol. 2010 Aug;39(8):817-20. doi: 10.1007/s00256-010-0879-3. Epub 2010**

**Feb 19. PubMed PMID: 20169344.**

**49: Sokolov M, Yaffe D, Ophir D. Retropharyngeal calcific tendinitis. Isr Med**

**Assoc J. 2009 Nov;11(11):701-2. PubMed PMID: 20108562.**

**50: Siwiec RM, Kushner DJ, Morrison JL. Retropharyngeal calcific tendinitis. J**

**Rheumatol. 2009 Jul;36(7):1546-7. doi: 10.3899/jrheum.081161. PubMed PMID:**

**19567634.**

**51: Park R, Halpert DE, Baer A, Kunar D, Holt PA. Retropharyngeal calcific**

**tendinitis: case report and review of the literature. Semin Arthritis Rheum. 2010**

**Jun;39(6):504-9. doi: 10.1016/j.semarthrit.2009.04.002. Epub 2009 Jun 21. PubMed**

**PMID: 19540570.**

**52: Offiah CE, Hall E. Acute calcific tendinitis of the longus colli muscle:**

**spectrum of CT appearances and anatomical correlation. Br J Radiol. 2009**

**Jun;82(978):e117-21. doi: 10.1259/bjr/19797697. PubMed PMID: 19451311.**

**53: Razon RV, Nasir A, Wu GS, Soliman M, Trilling J. Retropharyngeal calcific**

**tendonitis: report of two cases. J Am Board Fam Med. 2009 Jan-Feb;22(1):84-8.**

**doi: 10.3122/jabfm.2009.01.080034. PubMed PMID: 19124639.**

**54: Bladt O, Vanhoenacker R, Bevernage C, Van Orshoven M, Van Hoe L, D'Haenens P.**

**Acute calcific prevertebral tendinitis. JBR-BTR. 2008 Jul-Aug;91(4):158-9. PubMed**

**PMID: 18817090.**

**55: Kanzaria H, Stein JC. A severe sore throat in a middle-aged man: calcific**

**tendonitis of the longus colli tendon. J Emerg Med. 2011 Aug;41(2):151-3. doi:**

**10.1016/j.jemermed.2008.01.016. Epub 2008 Sep 23. PubMed PMID: 18815000.**

**56: Leep Hunderfund AN, Robertson CE, Bell ML, Busby DJ, Koehler TF, Ireland SP.**

**Calcific retropharyngeal tendinitis: unusual cause of acute neck pain with nuchal**

**rigidity. Neurology. 2008 Sep 2;71(10):778. doi:**

**10.1212/01.wnl.0000324917.44950.8b. PubMed PMID: 18765656.**

**57: Borrmann A, Niedermeyer HP, Arnold W. [Retropharyngeal tendinitis--a rare**

**differential diagnosis of retropharyngeal abscess]. Laryngorhinootologie. 2008**

**Mar;87(3):186-9. doi: 10.1055/s-2007-966887. German. PubMed PMID: 18415962.**

**58: Ellika SK, Payne SC, Patel SC, Jain R. Acute calcific tendinitis of the**

**longus colli: an imaging diagnosis. Dentomaxillofac Radiol. 2008 Feb;37(2):121-4.**

**doi: 10.1259/dmfr/23211511. PubMed PMID: 18239041.**

**59: Omezzine SJ, Hafsa C, Lahmar I, Driss N, Hamza H. Calcific tendinitis of the**

**longus colli: diagnosis by CT. Joint Bone Spine. 2008 Jan;75(1):90-1. Epub 2007**

**Aug 30. PubMed PMID: 17981487.**

**60: Van Kerkhove F, Geusens E, Knockaert D. Retropharyngeal calcific tendonitis.**

**Eur J Emerg Med. 2007 Oct;14(5):269-71. PubMed PMID: 17823562.**

**61: De Temmerman G, Marcelis S, Beeckman P. Acute calcific retropharyngeal**

**tendonitis. JBR-BTR. 2007 May-Jun;90(3):184-5. PubMed PMID: 17696088.**

**62: Kupferman TA, Rice CH, Gage-White L. Acute prevertebral calcific tendinitis:**

**a nonsurgical cause of prevertebral fluid collection. Ear Nose Throat J. 2007**

**Mar;86(3):164-6. PubMed PMID: 17427779.**

**63: Scutellari PN, Galeotti R, Leprotti S, Ridolfi M, Franciosi R, Antinolfi G.**

**The crowned dens syndrome. Evaluation with CT imaging. Radiol Med. 2007**

**Mar;112(2):195-207. Epub 2007 Mar 19. English, Italian. PubMed PMID: 17361376.**

**64: Jiménez S, Millán JM. Calcific retropharyngeal tendinitis: a frequently**

**missed diagnosis. Case report. J Neurosurg Spine. 2007 Jan;6(1):77-80. PubMed**

**PMID: 17233297.**

**65: Sanghvi DA, Jankharia BG, Purandare NC, Sundaram M. Acute calcific**

**retropharyngeal tendinitis. Orthopedics. 2006 Jul;29(7):561, 650-1. doi:**

**10.3928/01477447-20060701-17. PubMed PMID: 16866085.**

**66: Kusunoki T, Muramoto D, Murata K. A case of calcific retropharyngeal**

**tendinitis suspected to be a retropharyngeal abscess upon the first medical**

**examination. Auris Nasus Larynx. 2006 Sep;33(3):329-31. Epub 2006 Jan 24. PubMed**

**PMID: 16439087.**

**67: Chung T, Rebello R, Gooden EA. Retropharyngeal calcific tendinitis: case**

**report and review of literature. Emerg Radiol. 2005 Nov;11(6):375-80. Epub 2005**

**Jul 15. Review. PubMed PMID: 16344978.**

**68: Mihmanli I, Karaarslan E, Kanberoglu K. Inflammation of vertebral bone**

**associated with acute calcific tendinitis of the longus colli muscle.**

**Neuroradiology. 2001 Dec;43(12):1098-101. PubMed PMID: 11792053.**

**69: Szelei N, Tassart M, Le Breton C, Périé S, Boumenir ZE, Bazot M, Kadi N,**

**Bigot JM. [Calcific retropharyngeal tendinitis: unusual diagnosis]. J Radiol.**

**2001 Sep;82(9 Pt 1):1001-4. French. PubMed PMID: 11591929.**

**70: Eastwood JD, Hudgins PA, Malone D. Retropharyngeal effusion in acute calcific**

**prevertebral tendinitis: diagnosis with CT and MR imaging. AJNR Am J Neuroradiol.**

**1998 Oct;19(9):1789-92. PubMed PMID: 9802506.**

**71: Ring D, Vaccaro AR, Scuderi G, Pathria MN, Garfin SR. Acute calcific**

**retropharyngeal tendinitis. Clinical presentation and pathological**

**characterization. J Bone Joint Surg Am. 1994 Nov;76(11):1636-42. PubMed PMID:**

**7962023.**

**72: Chaimoff C, Bar-Ziv Y. [Calcific retropharyngeal tendinitis]. Harefuah. 1989**

**Jun 1;116(11):579-81. Hebrew. PubMed PMID: 2792934.**

**73: Artenian DJ, Lipman JK, Scidmore GK, Brant-Zawadzki M. Acute neck pain due to**

**tendonitis of the longus colli: CT and MRI findings. Neuroradiology.**

**1989;31(2):166-9. Review. PubMed PMID: 2664554.**

**74: Blome SA. Retropharyngeal calcific tendinitis. Australas Radiol. 1987**

**May;31(2):142-3. PubMed PMID: 3632521.**

**75: Fahlgren H. Retropharyngeal tendinitis. Cephalalgia. 1986 Sep;6(3):169-74.**

**PubMed PMID: 3768950.**

**76: Benanti JC, Gramling P, Bulat PI, Chen P, Lundstrom G. Retropharyngeal**

**calcific tendinitis: report of five cases and review of the literature. J Emerg**

**Med. 1986;4(1):15-24. PubMed PMID: 3461065.**

**77: Widlus DM. Calcific tendonitis of the longus colli muscle: a cause of**

**atraumatic neck pain. Ann Emerg Med. 1985 Oct;14(10):1014-7. PubMed PMID:**

**4037468.**

**78: Sarkozi J, Fam AG. Acute calcific retropharyngeal tendinitis: an unusual**

**cause of neck pain. Arthritis Rheum. 1984 Jun;27(6):708-10. PubMed PMID: 6732887.**

**79: Herwig SR, Gluckman JL. Acute calcific retropharyngeal tendonitis. Arch**

**Otolaryngol. 1982 Jan;108(1):41-2. PubMed PMID: 7053748.**

**80: Karasick D, Karasick S. Calcific retropharyngeal tendinitis. Skeletal Radiol.**

**1981;7(3):203-5. PubMed PMID: 7330678.**

**81: Haun CL. Retropharyngeal tendinitis. AJR Am J Roentgenol. 1978**

**Jun;130(6):1137-40. PubMed PMID: 418653**

15: Mills MK, Shah LM. Imaging of the perivertebral space. Radiol Clin North Am.

2015 Jan;53(1):163-80. doi: 10.1016/j.rcl.2014.09.008. Epub 2014 Oct 11. Review.

PubMed PMID: 25476179.

19: Riascos R, Lazor J, Vu L, Von Rischtl R, Zamudio R. Calcific tendinitis of

the prevertebral space: a case report. Ear Nose Throat J. 2013 Sep;92(9):E25-8.

PubMed PMID: 24057912.

21: Pellicer García V, Pérez Moya C, Magán Martín A. [Acute calcific prevertebral

tendinitis: case report and literature review]. Rev Esp Cir Ortop Traumatol. 2012

Sep-Oct;56(5):389-92. doi: 10.1016/j.recot.2012.05.009. Epub 2012 Jul 11. Review.

Spanish. PubMed PMID: 23594896.

22: Paik NC, Lim CS, Jang HS. Tendinitis of longus colli: computed tomography,

magnetic resonance imaging, and clinical spectra of 9 cases. J Comput Assist

Tomogr. 2012 Nov-Dec;36(6):755-61. doi: 10.1097/RCT.0b013e318269880c. PubMed

PMID: 23192216.

24: Schramm D, Glien A, Kösling S. [Prevertebral tendinitis - a rare differential

diagnosis of parapharyngeal abscess]. Rofo. 2013 Feb;185(2):167-9. doi:

10.1055/s-0032-1325438. Epub 2012 Oct 29. German. PubMed PMID: 23108900.

25: Wakabayashi Y, Hori Y, Kondoh Y, Asano T, Yamada A, Yamashita M, Nagatomi H.

Acute calcific prevertebral tendonitis mimicking tension-type headache. Neurol

Med Chir (Tokyo). 2012;52(9):631-3. Review. PubMed PMID: 23006873.

27: Hammer GP, Vollmann R, Tomazic PV, Simbrunner J, Friedrich G. Prevertebral

tendinitis: how to avoid unnecessary surgical interventions. Laryngoscope. 2012

Jul;122(7):1570-4. doi: 10.1002/lary.23338. Epub 2012 May 1. PubMed PMID:

22549265.

28: Lehner R, Stoupis C, Roth T, Andreisek G, Tamborrini G. [Rare cause of acute

neck pain]. Praxis (Bern 1994). 2011 Nov 2;100(22):1371-3. doi:

10.1024/1661-8157/a000716. German. PubMed PMID: 22048914.

31: Vollmann R, Hammer G, Simbrunner J. Pathways in the diagnosis of prevertebral

tendinitis. Eur J Radiol. 2012 Jan;81(1):114-7. doi: 10.1016/j.ejrad.2011.02.061.

Epub 2011 Mar 24. PubMed PMID: 21439752.

33: Vollmann R, Bohlsen D, Simbrunner J. [Benign prevertebral edema as expression

of retropharyngeal tendinitis--MRI image of a rare entity]. Rofo. 2010

Sep;182(9):809-10. doi: 10.1055/s-0029-1245491. Epub 2010 Jun 24. German. PubMed

PMID: 20577940.

35: Khurana B. Calcific tendinitis mimicking acute prevertebral abscess. J Emerg

Med. 2012 Jan;42(1):e15-6. doi: 10.1016/j.jemermed.2009.08.046. Epub 2009 Nov 17.

PubMed PMID: 19926427.

41: Harnier S, Kuhn J, Harzheim A, Bewermeyer H, Limmroth V. Retropharyngeal

tendinitis: a rare differential diagnosis of severe headaches and neck pain.

Headache. 2008 Jan;48(1):158-61. Epub 2007 Sep 12. PubMed PMID: 17868355.

44: Heckmann JG, Tröscher-Weber R, Pawlowski M, Seifert F, Lang CJ, Dörfler A,

Schwab S. [Retropharyngeal tendinitis. Differential diagnosis in the management

of acute neck pain]. Nervenarzt. 2006 Aug;77(8):952-7. German. PubMed PMID:

16832694.

46: Kuhn J, Harzheim A, Hartmann-Klosterkoetter U, Bewermeyer H. [Acute

calcifying prevertebral tendinitis of the M. longus colli as a rare cause of

intense neck and occipital lobe pain--a radiological imaging diagnosis]. Rofo.

2005 Apr;177(4):577-9. German. PubMed PMID: 15838766.

50: Kogstad O, Gjennestad AL, Svendsen D, Gåskjenn H. [Retropharyngeal tendinitis

after chiropractor therapy]. Tidsskr Nor Laegeforen. 1989 Jun

30;109(19-21):2001-3. Norwegian. PubMed PMID: 2749688.

53: Kaplan MJ, Eavey RD. Calcific tendinitis of the longus colli muscle. Ann Otol

Rhinol Laryngol. 1984 May-Jun;93(3 Pt 1):215-9. PubMed PMID: 6732105.

54: Weinberg S, Scott RA. Retropharyngeal tendinitis. Laryngoscope. 1982

Feb;92(2):181-2. PubMed PMID: 7162314.
